# β-Cell pre-mir-21 induces dysfunction and loss of cellular identity by targeting transforming growth factor beta 2 (*Tgfb2*) and Smad family member 2 *(Smad2)* mRNAs

**DOI:** 10.1016/j.molmet.2021.101289

**Published:** 2021-07-09

**Authors:** Sara Ibrahim, Macey Johnson, Clarissa Hernandez Stephens, Jerry Xu, Rachel Moore, Andrea Mariani, Christopher Contreras, Farooq Syed, Raghavendra G. Mirmira, Ryan M. Anderson, Emily K. Sims

**Affiliations:** 1 Department of Biochemistry and Molecular Biology, USA; 2 Center for Diabetes and Metabolic Diseases, USA; 3 Department of Pediatrics, USA; 4 Kovler Diabetes Center and the Department of Medicine, The University of Chicago, Chicago, IL, 60637, USA; 5 Wells Center for Pediatric Research, USA

**Keywords:** β-cell, Islet, microRNA 21, Dedifferentiation, Identity, β-cell dysfunction

## Abstract

**Objective:**

β-cell microRNA-21 (miR-21) is increased by islet inflammatory stress but it decreases glucose-stimulated insulin secretion (GSIS). Thus, we sought to define the effects of miR-21 on β-cell function using *in vitro* and *in vivo* systems.

**Methods:**

We developed a tetracycline-on system of pre-miR-21 induction in clonal β-cells and human islets, along with transgenic zebrafish and mouse models of β-cell-specific pre-miR-21 overexpression.

**Results:**

β-cell miR-21 induction markedly reduced GSIS and led to reductions in transcription factors associated with β-cell identity and increased markers of dedifferentiation, which led us to hypothesize that miR-21 induces β-cell dysfunction by loss of cell identity. *In silico* analysis identified transforming growth factor-beta 2 (*Tgfb2*) and Smad family member 2 *(Smad2)* mRNAs as predicted miR-21 targets associated with the maintenance of β-cell identity. *Tgfb2* and *Smad2* were confirmed as direct miR-21 targets through RT-PCR, immunoblot, pulldown, and luciferase assays. *In vivo* zebrafish and mouse models exhibited glucose intolerance, decreased peak GSIS, decreased expression of β-cell identity markers, increased insulin and glucagon co-staining cells, and reduced *Tgfb2* and *Smad2* expression.

**Conclusions:**

These findings implicate miR-21-mediated reduction of mRNAs specifying β-cell identity as a contributor to β-cell dysfunction by the loss of cellular differentiation.

## Highlights

• *In vitro* and *in vivo* β-cell miR-21 induction reduced glucose-stimulated insulin secretion and led to hyperglycemia.
• β-cell miR-21-induction reduced transcription factors linked to β-cell identity and increased markers of dedifferentiation.
• miR-21 mediated effects on β-cell identity and function were mediated in part by direct targeting of *Tgfb2* and *Smad2* mRNAs.

## Abbreviations

microRNA: (miRNA)
Type 1 Diabetes: (T1D)
Type 2 Diabetes: (T2D)
programmed cell death 4: (PDCD4)
B Cell Lymphoma 2: *(Bcl2*)
Transforming Growth Factor Beta 2: (*Tgfb2*)
SMAD Family Member 2: (*Smad2*)
Glucose stimulated insulin secretion: (GSIS)
locked nucleic acid: (LNA)
quantitative real-time PCR: (qRT-PCR)
intraperitoneal glucose tolerance tests: (IPGTTs)
untranslated region: (UTR)
MAF BZIP Transcription Factor A: (*Mafa)*
NK6 Homeobox 1: (*Nkx6.1)*
Insulin 1 and 2: *(Ins1* and *Ins2)*
Neuronal Differentiation 1: (*Neurod1)*
Solute Carrier Family 2 Member 2: (*Glut2)*
Proprotein Convertase Subtilisin/Kexin Type 1: (*Pcsk1)*
Proprotein Convertase Subtilisin/Kexin Type 1: (*Pcsk2)*
Neurogenin 3: (*Ngn3*)
Nanog Homeobox: (*Nanog*)
L-Myc-1 Proto-Oncogene: (*L-Myc*)
Aldehyde dehydrogenase 1a3: (*Aldh1a3)*
heat-shock inducible β-cell miR-21 transgenic fish: (Tg(*HS βmiR-21*))
tamoxifen-inducible β-cell–specific transgenic mice: (Tg(*βmiR-21*))

## 1 Introduction

With a prevalence of 30.2 million people in the US alone, diabetes poses a tremendous domestic and international health burden [1]. A commonality between both type 1 diabetes (T1D) and type 2 diabetes (T2D) is reduced functional β-cell mass; either in association with autoimmune β-cell destruction (T1D) or with prolonged exposure to insulin resistance, systemic elevations in proinflammatory cytokines, and saturated free fatty acids (T2D) [2]. In both T1D and T2D, β-cells may exhibit maladaptive signaling responses to inflammatory stress, potentially exacerbating β-cell dysfunction and death or accelerating β-cell autoimmune destruction [3]. An improved understanding of these molecular signaling pathways may pave the way for novel therapies targeting β-cell dysfunction before or after diabetes development.

MicroRNAs (miRNAs) are small RNA molecules that classically repress translation through either direct inhibition or mRNA destabilization [4]. Islet miRNA expression profiling and analyses have identified multiple β-cell miRNAs as critical regulators of β-cell differentiation, development, death, function, and as mediators of the complex β-cell response to inflammatory stress [4, 5]. This work has identified that β-cell miR-21-5p is increased in models of inflammation and diabetes [[6], [7], [8], [9], [10]]. The role of miR-21-5p induction in β-cells has been studied by several groups. Several studies have shown that β-cell miR-21-5p targets the pro-apoptotic protein programmed cell death 4 (PDCD4) [7, 11], and that direct reductions in PDCD4 *in vivo* lead to an increase in β-cell viability [11]. Inhibition of miR-21-5p *in vitro* reduced mouse insulinoma (MIN6) cell death [7], but overexpression using lentiviral transduction has also been shown to increase β-cell death, reduce the β-cell number, and increase cell proliferation [12]. Our group showed that miR-21-5p mimic transfection increased β-cell death by inhibition of the pro-survival mRNA B cell lymphoma 2 *(Bcl2)*, despite decreased PDCD4 [6]. Using RNA duplexes or mimic transfection, several groups have also shown a negative effect of miR-21-5p overexpression on GSIS [6, 7] and *in vitro* inhibition, using a miR-21 inhibitor improved insulin release from cytokine-treated MIN6 cells [7]. However, more comprehensive studies identifying mechanisms of pre-miR-21's effects on β-cell function and the *in vivo* roles of β-cell miR-21 are required.

To bridge this knowledge gap, we developed an *in vitro* lentiviral model to define the effects of β-cell pre-miR-21 (hereafter referred to as miR-21) induction at levels comparable to those observed in models of islet inflammatory stress [6]. This model demonstrated that miR-21 induction reduced insulin secretion in concert with the expression of key transcription factors associated with β-cell identity. Based on a target prediction analysis, we hypothesized that miR-21 induces β-cell dysfunction by the inhibition of mRNAs critical for β-cell function and identity: transforming growth factor-beta 2 (*Tgfb2*) and Smad family member 2 (*Smad2*) mRNAs in the *Tgfb2* pathway. *Tgfb2* is a member of the TGF-β superfamily of proteins that is involved in diverse roles across different cell types by signaling through a group of transcription factors called Smads [13]. Specifically, *Tgfb2* has been shown to play an important role in β-cell identity and function [14]. To further test the roles of β-cell miR-21 *in vivo*, we developed zebrafish and mouse models of inducible β-cell-specific miR-21 overexpression. Our results implicate miR-21 as a regulator of β-cell identity in part, by direct targeting of *Tgfb2* and *Smad2* mRNAs.

## 2 Methods

### 2.1 Lentiviral miR-21 induction

The rat pre-miR-21 and a scrambled miR-21 sequence were cloned into a pInducer lentiviral vector (Gibson cloning; Addgene plasmid #44012). Viral particles were concentrated for INS1 823/13 transduction to generate INS1-miR-21 and INS1-scramble cells [15]. Based on dose–response experiments (Supplementary Figure 2), 48 h 5 μg/ml doxycycline was used for miR-21 induction.

### 2.2 Cell transfection

4 × 10^5^ cells/well were treated for 48 h with 100 pmol of a miR-21 locked nucleic acid (LNA) inhibitor (Exiqon), or negative controls (Qiagen), or 1.25 μg of a *Tgfb2* vector (OriGene) complexed with 3 μl Lipofectamine 3000 and 100 μl Opti-MEM (ThermoFisher). LNA-transfected cells were treated with 5 ng/ml IL-1β for 24 h. The inhibitor was validated by confirming the increase in expression levels of previously validated targets *Bcl2* and *Pdcd4* (Supplementary Figure 3).

### 2.3 Islet MiR-21 predicted target analysis

*In silico* analysis was performed to identify predicted miR-21-5p or -3p targets overlapping with human islet mRNAs downregulated under conditions of inflammatory stress and diabetes (workflow described in detail in Supplementary Figure 1 and identified targets listed in Supplementary File 1) [[16], [17], [18], [19], [20], [21], [22], [23], [24], [25]].

### 2.4 RNA sequencing

Isolated RNA was used to prepare dual-indexed non stranded cDNA libraries using SMART- Seq v4 Ultra Low Input RNA Kit (Clontech) [26]. mRNA sequencing was performed with greater than 20 million reads per sample. Libraries were sequenced with a HiSeq 4000 system (Illumina).

### 2.5 Other *in vitro* assays

Cytokine treatment of cells was performed with 5 ng/ml IL1β from R&D systems. RNA isolation and reverse transcription followed by quantitative real-time PCR (qRT-PCR) were performed (Qiagen miScript system) [6]. miRNA or mRNA expression was quantified relative to U6 or β-actin, respectively, using the comparative Ct method (Primer sequences in Supplementary Table 1) [6]. Pulldown of mRNAs bound to 50 nM biotinylated miR-21-3p, miR-21-5p, or control *Caenorhabditis elegans* miR-67 was performed as described [27]. Luciferase assays were performed using a Gaussia luciferase/secreted alkaline phosphatase dual reporter system (GeneCopoeia) and wild-type rat *Tgfb2* 3′ untranslated region (UTR) and *Smad2* 3′UTR or mutated 3′UTRs for *Tgfb2* (positions 1281–1289) and *Smad2* (positions 8900–8908) [6]. Immunoblotting was performed as described, visualized using an Odyssey imaging system, and quantified by LI-COR software (LI-COR Biotech) (antibodies in Supplementary Table 2) [6].

Static GSIS and perifusion were performed as described with supernatants assayed for insulin using ELISA (Cisbio) and normalized to total DNA content (PICO Green Assay; Invitrogen) [28, 29].

### 2.6 MiR-21 induction in zebrafish

All animal experiments were carried out in accordance with the National Institutes of Health guide for the care and use of laboratory animals. To generate *Tg*(*hs:CS-βmiR-21*) zebrafish, a zebrafish pre-miR-21 amplicon was put in place of the H2B-GFP coding sequence contained in the transgenesis vector used to make the Tg(*hs:CSH*) transgenic line. This was generated using high-fidelity PCR, followed by subcloning to a site downstream of the lox-mCherry-STOP-lox cassette. *Tg*(*hs:CS-βmiR-21*) fish were intercrossed with *Tg*(*ins:Cre*)^*s924*^ fish [30] to generate fish exhibiting heat-shock inducible miR-21 overexpression, specifically within β-cells. Embryos were heat-shocked for 10 min at 39 °C. RNA from 15 embryos/clutch and 20 islets/clutch was used for PCR analysis. Glucose colorimetric assays (Bio Vision #K686) were performed using 20 embryos/clutch. Zebrafish embryos were fixed with 3% formaldehyde in PEM buffer at 4 °C overnight and deyolked for immunostaining. Glucose colorimetric assays (BioVision #K686) were performed using 20 embryos/clutch.

### 2.7 Tg(*βmiR-21)* mice

Tg(CAG-Z-*miR-21*-EGFP) [31] mice (backcrossed on a C57BL6/J background for >10 generations) were crossed with *Ins1tm1(CreERT2)*Thor [32] mice to generate Tg(*βmiR-21)* mice. Eight-week Tg(*βmiR-21)* mice and littermate controls (Cre+ and Cre-) were treated with 1 mg/day x 6 days intraperitoneal (IP) tamoxifen. IP glucose tolerance tests (IPGTTs) were performed 21-days post tamoxifen injection after overnight fast, using 2 g/kg body weight of glucose [28]. Tail vein glucose was determined (AlphaTRAK glucometer; Abbott) at 0, 10, 20, 30, 60, 90, and 120-min post injection. Insulin sensitivity was measured with IP insulin tolerance testing (IPITT) after a 2-h fast, using IP injection of 0.75 U/kg bodyweight of regular humulin-R insulin (Eli Lilly) [28]. Tail vein glucose was measured at 0, 10, 20, 30, and 60min post injection. Islets were isolated using collagenase 28 days after the initial tamoxifen injection [33].

### 2.8 Immunofluorescence

Zebrafish embryos and mouse pancreata were fixed and immunostained as described [30].

Primary antibodies (Supplementary Table 2) were detected with 1:500 dilutions of Alexa-conjugated secondary antibodies (Jackson ImmunoResearch). Confocal imaging was performed using a Zeiss LSM700 microscope and quantified by measuring pixel density per insulin-positive cell (Fiji software). To measure nuclear vs. cytoplasmic intensity of markers in cells, a DAPI signal was used as a mask to quantify only pixel density within the nucleus. To quantify Insulin^+^ glucagon^+^ polyhormonal cells, all visible individual islet cells that exhibited both insulin staining and glucagon staining were counted.

### 2.9 Human islet transduction

Human islets were obtained from the IIDP [6]. Dispersed cells from 300 islets were transduced with 50 μl of concentrated lentivirus as above, and then treated with 10 μg/ml of doxycycline for 48 h followed by a 24 h recovery period.

### 2.10 Statistical analysis

Statistical analyses were performed using GraphPad Prism Version 7.1 (GraphPad software). Data are presented as means ± standard error of the mean (SEM). Student's t-tests or Kolmogorov–Smirnov tests were used for comparison between the experimental and control groups as indicated. One-way ANOVA with Tukey's post-test for multiple comparisons was used when comparing >2 groups. A p-value of ≤0.05 was considered significant

### 2.11 Data and resource availability

The datasets generated and/or analyzed during the present study are included in the published article (and its online supplementary files).

## 3 Results

### 3.1 Induction of miR-21 in INS1 β-cells leads to β-cell dysfunction and loss of identity

**Figure 1:**
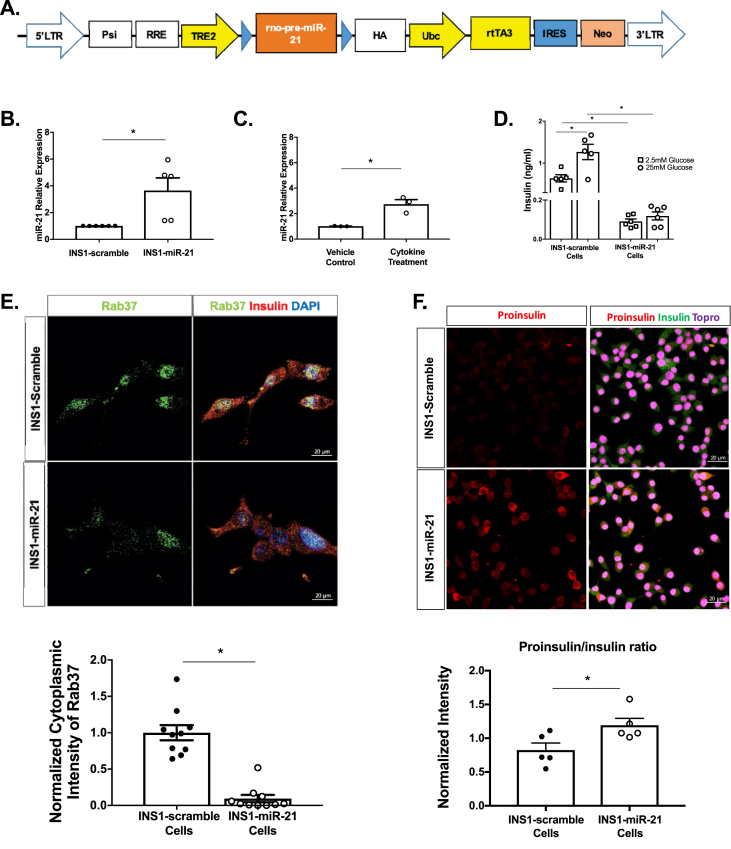
**β-cell miR-21 induction results in impaired function. (A)** Construct for the miR-21 overexpressing lentiviral system (LTR=long terminal repeat, Psi=packaging signal, RRE=rev response element, TRE2=tetracycline response element, Ubc=ubiquitin, HA=tag protein, rtTA3=reverse tetracycline trans-activator, IRES=internal ribosome entry site, Neo=Neomycin cassette). **(B)** miR-21 induction in 48 h 5 ug/ml doxycycline-treated INS1-miR-21 cells vs. INS1-scramble control cells. **(C)** miR-21 induction following 24 h of 5 ng/ml IL1β treatment in INS1 cells. **(D)** Baseline and glucose-stimulated insulin secretion were decreased in INS1-miR-21 cells as compared to INS1-scramble cells. **(E)** Cytoplasmic staining for the granule marker Rab37 (a marker of secretory granules) was decreased in INS1-miR-21 cells. **(F)** Proinsulin to insulin ratio was increased in INS1-miR-21 cells based on quantification of staining for proinsulin and insulin. Signal intensity from experimental images was normalized to signal intensity from control images, giving a relative expression for all cell staining experiments. n = 3–12 (4–5 transductions of cells); ∗p < 0.05.

Because miRNA mimic transfection leads to supraphysiologic increases in miRNAs [34], we generated a tetracycline-on, doxycycline dependent system of lentiviral pre-miR-21 (hereafter referred to as miR-21) induction to define the effect of smaller fold increases in β-cell miR-21 (Figure 1A). This system allowed for pre-miR-21 induction at a relative expression more comparable to that of β-cells or islets treated with proinflammatory cytokines (Figure 1B–C) [6]. Compared to INS1-scramble cells, INS1-miR-21 cells exhibited a pronounced reduction in insulin secretion at baseline and a response to high glucose (Figure 1D). Quantification of cytoplasmic Rab37 as a marker of secretory granules demonstrated reduced staining in INS1-miR-21 cells compared to scramble controls (Figure 1E). Staining and quantification of proinsulin to insulin express expression showed an increase in the ratio of immature proinsulin to mature insulin in INS1-miR-21 cells as compared to INS1-scramble control cells (Figure 1F).

**Figure 2:**
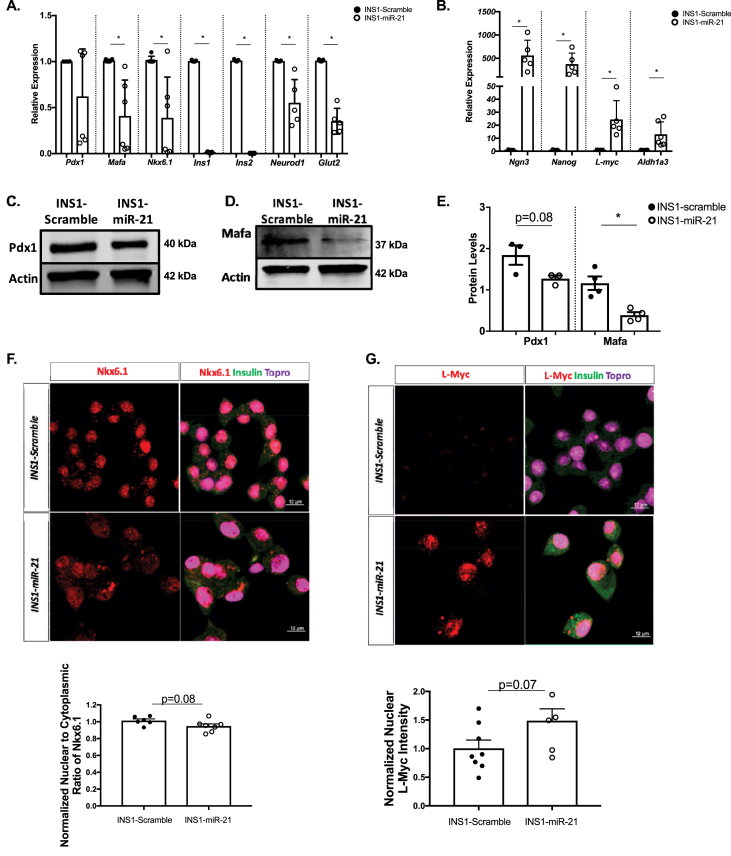
**RT-PCR analysis and staining of INS1-miR-21 cells identify reduced expression of genes impacting β-cell function and identity. (A)** The qRT-PCR analysis demonstrated that transcripts for *MafA, Nkx6.1, Ins1, Ins2, Neurod1,* and *Glut2,* were all decreased after miR-21 induction in INS1-miR-21 cell lines. **(B)** The qRT-PCR analysis determined that transcripts for β-cell progenitor markers *Ngn3, Nanog, L-myc,* and a gene associated with β-cell dysfunction in the context of dedifferentiation, *Aldh1a3*, were all increased after miR-21 induction in INS1-miR-21 cell lines. Western blot analysis and quantification demonstrated a trend toward a decrease in the levels of Pdx1 and significantly decreased levels of MafA **(C–E)**. **(F)** Staining for Nkx6.1 showed a trend towards a decrease in the nuclear to cytoplasmic ratio in INS1-miR-21 cells as compared to controls. **(G)**Staining for L-Myc demonstrated a trend toward an increase in nuclear expression in INS1-miR-21 cell lines. n = 3–9 (3–4 transductions of cells); ∗p < 0.05.

Recent data have identified β-cell dedifferentiation, loss of identity, or reversion to a progenitor-like state, as a compensatory response to islet inflammatory stress, with evidence of β-cell dedifferentiation in models of T1D and T2D [35]. To test whether our observed phenotype could be associated with this phenomenon, RT-PCR was performed to validate changes in gene expression associated with loss of β-cell identity *in vitro*. We first assessed transcription factors classically associated with β-cell identity and function (Figure 2A). Here, miR-21 induction decreased mRNA expression of MAF BZIP transcription factor A (*Mafa),* NK6 homeobox 1 (*Nkx6.1),* both insulin genes *(Ins1* and *Ins2),* neuronal differentiation 1 (*Neurod1),* and solute carrier family 2 member 2 (*Glut2).* We also performed western blots to test changes in protein expression with miR-21 induction. Here, we measured a trend toward decreased protein expression level of Pdx1 and a significant decrease in MafA (Figure 2C–E). No significant decrease in protein levels of Glut2 was measured, as detected by western blot analysis (Supplementary Figure 4A). Immunostaining was also performed to measure decreases in protein levels of transcription factors associated with β-cell identity and function, which demonstrated a trend toward decreased nuclear (relative to cytoplasmic) levels of Nkx6.1 (Figure 2F). No decrease in urocortin staining was measured (Supplementary Figure 4B).

We next assessed markers associated with dedifferentiation and β-cell progenitor markers. Consistent with a shift towards a more progenitor-like state, we observed increased neurogenin 3 (*Ngn3*), Nanog homeobox (*Nanog*), L-myc 1 proto-oncogene (*L-Myc*), and aldehyde dehydrogenase 1a3 (*Aldh1a3)* expression after miR-21 induction (Figure 2B). To test if observed effects of miR-21 overexpression on β-cell function were associated with an increase in protein levels of β-cell progenitors, we performed immunostaining for the β-cell progenitor marker L-myc (Figure 2G), which showed a trend towards increased nuclear expression in INS1-miR-21 cells as compared to scramble control cells.

To determine global transcript changes induced by the induction of miR-21, we performed RNA sequencing of transduced INS1-miR-21 cells and INS1-scramble control cells. Multidimensional scaling (MDS) analysis demonstrated that INS1-miR-21 samples exhibited a substantially different genetic profile compared to the INS1-scramble control samples (Supplementary Figure 5A–B), with differential expression data in (Supplementary File 2). Network analysis (Supplementary Figure 5C) suggested that increases in miR-21 were associated with downregulation of genes associated with β-cell differentiation and identity, along with genes involved in β-cell function, such as glucose metabolism and insulin secretion.

### 3.2 miR-21 target analysis identifies *Tgfb2* and *Smad2* as direct mRNA targets involved in β-cell commitment

**Figure 3:**
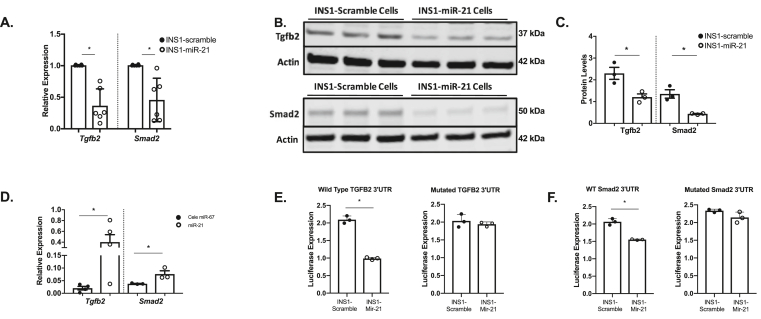
**INS1-miR-21 cells directly target the *Tgfb2* pathway. (A)** The qRT-PCR analysis demonstrated that transcripts for *Tgfb2* and *Smad2* were decreased after miR-21 induction in INS1-miR-21 cells. **(B–C)** Western blot analysis showed decreased Tgfb2 and Smad2 protein expression in miR-21 induced INS1-miR-21 cells. **(D)***Tgfb2* and *Smad2* mRNAs were enriched in the miR-21 biotinylated pulldown samples normalized to cell lysate and compared to C-elegans control miR-67 biotinylated mimic pulldown. **(E–F)**: Luciferase assays showed that activity was decreased after miR-21 induction in cells expressing wild type 3′UTR, but not in cells expressing the mutated 3′UTR for **(E)***Tgfb2* and (F) *Smad2*. n = 3–6; ∗p < 0.05.

**Figure 4:**
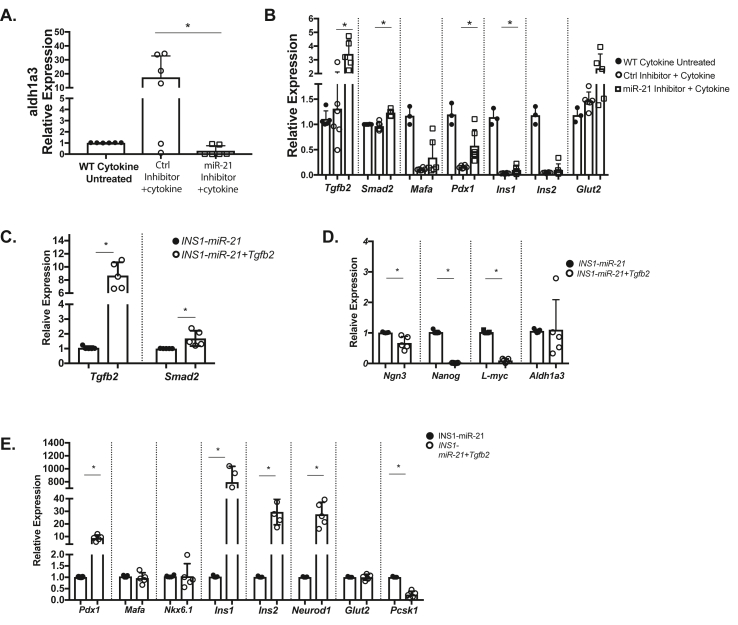
**Inhibition of miR-21 in INS1 cells blocks the effects of inflammatory cytokines on β-cell identity and overexpression of Tgfb2 ameliorates effects of miR-21 on β-cells**. Compared to 24-h treatment with IL1- β alone, pretreatment with a miR-21 inhibitor resulted in **(A)** reduced *Aldh1a3* expression. **(B)** Cytokine-induced reductions in *Tgfb2* and *Smad2, Pdx1,* and *Ins1* were also abrogated by pre treatment with a miR-21 inhibitor, with a trend toward an increase in levels of *MafA, Ins2,* and *Glut2*. Wild-type cytokine untreated results are shown for comparison, with statistical comparisons performed between the cytokine-treated control inhibitor and miR-21 inhibitor groups. **(C)** The qRT-PCR analysis demonstrated that transcripts for *Tgfb2* and *Smad2* are increased in INS1-miR-21 cells treated with a *Tgfb2* overexpression vector as compared to INS1-miR-21 control cells. **(D)** Overexpression of Tgfb2 resulted in decreased expression of β-cell progenitor markers *Ngn3, Nanog,* and *L-myc* in INS1-miR-21 cells. **(E)** Overexpression of *Tgfb2* also resulted in increased expression of *Pdx1, Ins1, Ins2,* and *Neurod1*. n = 3–5; ∗p < 0.05.

To further probe molecular pathways and identify potential direct mRNA targets impacted by increased islet miR-21 during diabetes development, we performed an analysis of predicted mRNA targets of miR-21-5p and 3p using target prediction software, and overlapped these results with mRNAs reduced in publicly available sequencing datasets from human islets treated with cytokines or with T2D (Supplementary Figure 1). Notably, several members of the transforming growth factor-beta 2 (*Tgfb2)* pathway, including *Tgfb2* and *Smad2*, were identified as potential direct targets (Supplementary Figure 1B). Because this pathway has been implicated in the regulation of β-cell identity and commitment [14] and also because miR-21 is predicted to directly target several genes within the pathway, we chose to focus on *Tgfb2* and *Smad2* as potential direct mRNA targets that could contribute to observed effects of miR-21 induction on β-cell identity *in vitro*. Consistent with a negative effect of miR-21 on these mRNAs, RT-PCR analysis demonstrated a significant decrease in both *Tgfb2* and *Smad2* transcripts after miR-21 induction (Figure 3A). Immunoblot analysis also demonstrated decreased protein expression of both Tgfb2 and Smad2 after miR-21 induction (Figure 3, Figure 4).

To test whether *Tgfb2* and *Smad2* mRNAs directly bind to miR-21, we performed a streptavidin bead-based pulldown after transfection with biotinylated miR-21-5p, miR-21-3p, or cel-miR-67 duplex control construct. Compared to control pull-downs, *Tgfb2* and *Smad2* mRNAs were significantly enriched within the biotinylated miR-21 pulldown, suggesting direct binding to miR-21-5p and -3p, respectively (Figure 3D). To determine whether miR-21 leads to functional inhibition of *Tgfb2* and *Smad2* translation, we also performed luciferase reporter assays (Figure 3E–F). Here, consistent with functional inhibition, miR-21 overexpression reduced luciferase activity in constructs containing wild type 3′ UTRs for both *Tgfb2* and *Smad2*. By contrast, miR-21 had no effect on *Tgfb2* and *Smad2* 3’ UTRs with mutated predicted binding sites.

### 3.3 Inhibition of miR-21 or overexpression of Tgfb2 can partially abrogate reductions in mRNAs linked to β-cell identity

Islet inflammatory stress increases islet miR-21 expression and is associated with the altered β-cell identity [38]. To test whether miR-21 inhibition can block the effect of inflammatory cytokines on altered β-cell identity, we treated INS1 cells with a miR-21 inhibitor followed by a 24 h IL1β treatment. Consistent with a role for miR-21 in cytokine-induced β-cell dysfunction and loss of identity, treatment with cytokines increased *Aldh1a3* expression, but pretreatment with miR-21 inhibitors abrogated these cytokine-induced increases (Figure 4A). Additionally, compared to cytokine-treated cells alone, miR-21 inhibition increased expression of *Tgfb2*, *Smad2, Pdx1,* and *Ins1* mRNAs, with a trend toward an increase in *MafA, Ins2,* and *Glut2* mRNAs (Figure 4B). Expression levels in untreated wild-type cells are also shown for each transcript for comparison.

To further focus on the *Tgfb2* signaling pathway as a mechanistic etiology of miR-21's effects on β-cell identity, we overexpressed *Tgfb2* in miR-21 induced lentiviral cells to test if increased *Tgfb2* would reverse effects of miR-21 overexpression (Figure 4C). As *Smad2* is a downstream modulator in the *Tgfb2* pathway, this also resulted in increased *Smad2* expression. *Tgfb2* overexpression in INS1-miR-21 cells decreased expression of markers of β-cell dysfunction and dedifferentiation (Figure 4D). *Tgfb2* overexpression also increased the expression of multiple mRNAs associated with β-cell identity and function in miR-21 induced cells (Figure 4E).

### 3.4 β-Cell-specific miR-21 induction in zebrafish results in a phenotype of β-cell dysfunction and dedifferentiation in association with reduced *Tgfb2* and *Smad2* expression

**Figure 5:**
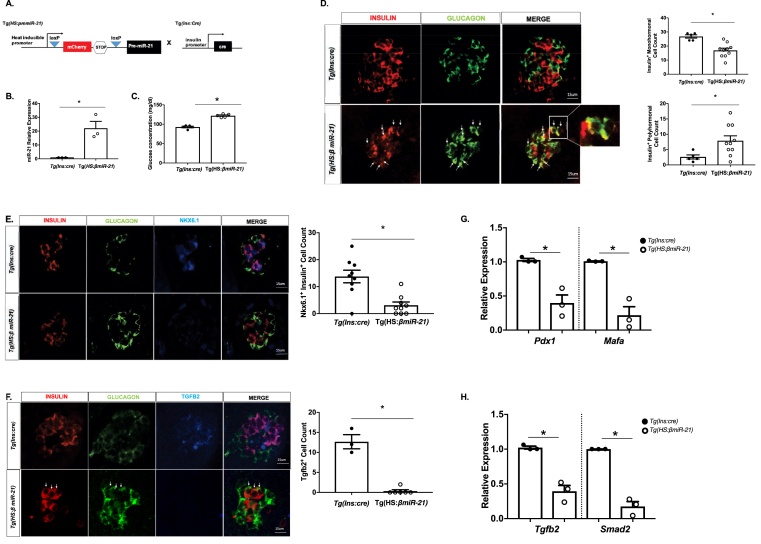
**A zebrafish model of β-cell-specific miR-21 induction exhibits hyperglycemia, increased bihormonal cells, decreased expression of β-cell identity markers, and reduced expression of Tgfb2 and Smad2 (A)** Construct for the Tg(*HS:miR-21*) line and breeding strategy to develop Tg(*HS:βmiR-21*) fish. In all experiments, Tg(*HS:βmiR-21*) tissues were compared to samples from clutch-mate Tg(*Ins:cre*) controls. **(B)** miR-21 levels are increased in Tg(*HS:miR-21*) islets. **(C)** Systemic glucose levels were increased after miR-21 induction. **(D)** A decrease in insulin^+^ cells and an increase in insulin^+^ glucagon^+^ co-positive cells (examples shown with white arrows) were observed in islets from miR-21 induced embryos. **(E)** Levels of Nkx6.1 are decreased within the nuclei of miR-21 induced larvae. **(F)** A decrease in Tgfb2^+^ cells was observed in islets of Tg(*HS:miR-21*) embryos. White arrows highlight insulin^+^ glucagon^+^ co-positive cells. **(G)** RT-PCR demonstrated reduced mRNA expression of *MafA* and *Pdx1* in islets isolated from Tg(*HS:miR-21*) embryos. **(H)** Expression of *Tgfb2* and *Smad2* mRNAs was decreased in islets isolated from Tg(*HS:miR-21*) embryos. RNA from at least 3 clutches, with 15 embryos/clutch and 20 islets/clutch was used for RT-PCR analysis. n=3–10; ∗p < 0.05.

To define the effects of β-cell miR-21 *in vivo*, we generated heat-shock inducible β-cell miR-21 transgenic fish (Tg(*HS:βmiR-21*)) (Figure 5A–B) by crossing *Tg(HS:pre-miR-21)* fish to *Tg(Ins:Cre)* fish. Tg(*HS:βmiR-21*) embryos exhibited hyperglycemia compared to controls (Figure 5C). Furthermore, reduced numbers of insulin^+^ β-cells were observed in Tg(*HS:βmiR-21*) islets (Figure 5D). Consistent with the loss of differentiation in association with miR-21 overexpression, Tg(*HS:βmiR-21*) islets also exhibited increased numbers of insulin^+^ glucagon^+^ co-staining cells, marked by white arrows (Figure 5D). Although immunostaining for genes associated with mature β-cell identity was limited by available antibodies exhibiting specific cross-reactivity with zebrafish antigens, we identified depletion of Nkx6.1, a key transcription factor in the maintenance of β-cell function and maturation, in insulin^+^ cells from Tg(*HS:βmiR-21*) islets (Figure 5E) [39]. Consistent with our data in INS1-miR-21 cells, Tgfb2 immunostaining was decreased in Tg(*HS:βmiR-21*) islets (Figure 5F). RT-PCR also showed reductions in *MafA* and *Pdx1* mRNAs in Tg(*HS:βmiR-21*) islets (Figure 5G). Although Smad2 antibodies were not available for immunofluorescence in zebrafish, both *Tgfb2* and *Smad2* mRNA levels were decreased in Tg(*HS:βmiR-21*) islets (Figure 5H).

### 3.5 β-cell MiR-21 induction in a mouse model leads to glucose intolerance and a phenotype consistent with loss of β-cell identity, in association with reduced Tgfb2 and Smad2 expression

**Figure 6:**
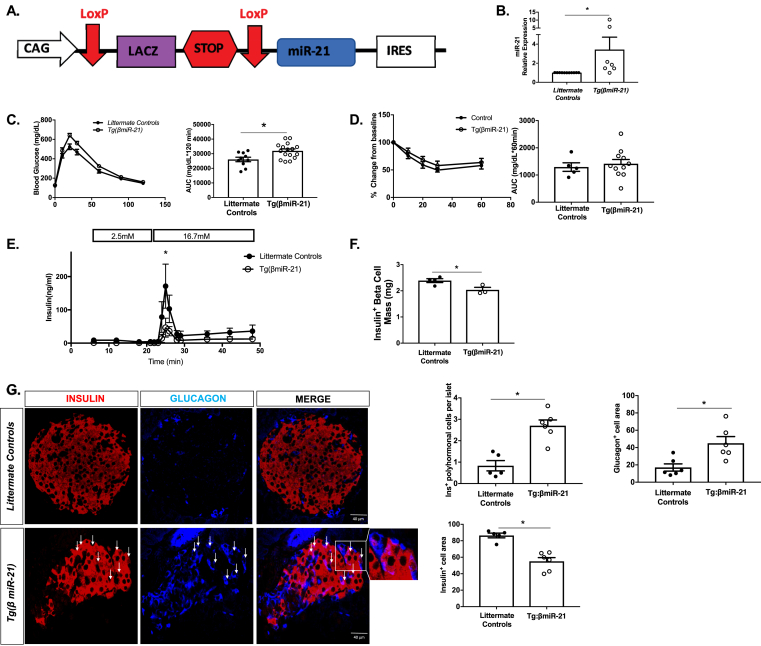
**Tg(*βmiR-21*) mice are glucose intolerant and display a phenotype of islet β-cell dysfunction and loss of identity (A)** Construct for Tg(*βmiR-21)* mice. For all experiments, tamoxifen-treated Tg(*βmiR-21)* mice were compared to tamoxifen-treated Tg(CAG-Z-*miR-21*-EGFP) mice and tamoxifen-treated *Ins1tm1(CreERT2)*Thor mice. **(B)** miR-21 levels are increased in islets from the Tg(*βmiR-21)* mice. **(C)** Glucose tolerance testing (GTT) showed that the Tg(*βmiR-21)* mice are glucose intolerant as compared to littermate controls. **(D)** Insulin tolerance testing (ITT) showed no differences between Tg(*βmiR-21)* mice and controls. **(E)***Ex vivo* perifusion analysis showed a significant decrease in peak insulin secretion in islets from Tg(*βmiR-21)* mice. **(F)** Immunohistochemistry analysis demonstrated decreased insulin + β-cell mass in Tg(*βmiR-21)* mice. **(G)** Tg(*βmiR-21)* islets exhibited increased insulin^+^ glucagon^+^ co-expressing cells (white arrows). Tg(*βmiR-21)* islets also demonstrated an increase in glucagon^+^ cell area and a decrease in insulin^+^ cell area. n = 5–15 for metabolic testing islet RT-PCR and n = 3–4 mice for histologic analyses; ∗p < 0.05.

To define the effects of β-cell miR-21 induction on glucose homeostasis in a mammalian system, we generated tamoxifen-inducible β-cell-specific transgenic (Tg(*βmiR-21*)) mice (Figure 6A–B). Compared to tamoxifen-treated littermate controls, Tg(*βmiR-21*) mice exhibited mild glucose intolerance on IPGTTs, without significant differences in insulin tolerance (Figure 6C–D). Similar results were verified in Cre^+^ versus Cre^−^ controls to rule out the effect of Cre on glucose tolerance (Supplementary Figure 6A). No changes in miR-21 expression were observed in other tissues tested (Supplementary Figure 6B). *Ex vivo* peak insulin secretion was decreased in Tg(*βmiR-21*) islets (Figure 6E). Insulin positive β-cell mass was also decreased in Tg(*βmiR-21*) mice (Figure 6F). Next, we assessed endpoints that could point to changes in β-cell identity. Similar to our zebrafish model, altered islet architecture with increased insulin^+^ glucagon^+^ co-staining cells was also observed in the Tg(*βmiR-21*) mice (white arrows) (Figure 6G). Additionally, Tg(*βmiR-21*) mice demonstrated an increase in glucagon^+^ cell area and a decrease in insulin+ cell area (Figure 6G).

**Figure 7:**
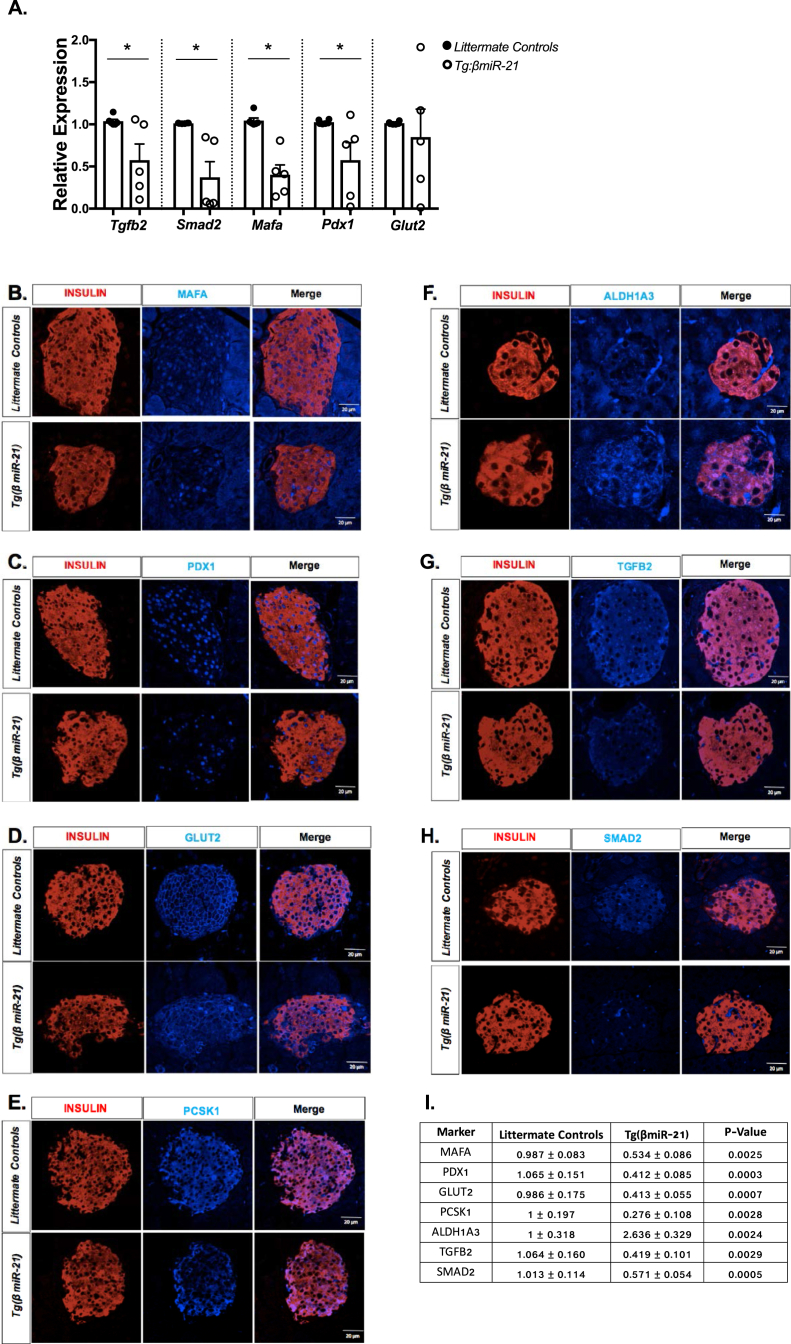
**Tg(*βmiR-21*) mouse islets display decreases in markers of β-cell identity.** For all experiments, tamoxifen-treated Tg(*βmiR-21)* mice were compared to tamoxifen-treated Tg(CAG-Z-*miR-21*) mice and tamoxifen-treated *Ins1tm1(CreERT2)*Thor mice. Transcript levels of markers essential for β-cell identity were measured by RT-PCR **(A)**. Decreased **(B)** MafA and **(C)** Pdx1 expression was quantified in nuclei of Insulin+ cells in Tg(*βmiR-21*) islets. **(D)** Decreased expression of Glut2 and Pcsk1 **(E)** were also observed in Tg(*βmiR-21*) islets. Levels of Aldh1a3 were increased in Tg(*βmiR-21*) mice as compared to littermate controls **(F)**. Decreased expression of Tgfb2 **(G)** and Smad2 **(H)** were seen in insulin+ cells in Tg(*βmiR-21*) islets. **(I)** Immunofluorescence quantification is displayed as mean ± SEM for littermate controls and Tg(*βmiR-21*) mice, respectively. Immunofluorescence intensity was quantified for 3 islets per mouse for n = 3–4 mice per group; ∗p < 0.05.

Consistent with phenotypes observed in Tg(*HS:βmiR-21*) zebrafish, RT-PCR analysis of Tg(*βmiR-21*) mouse islets showed decreased expression of *Tgfb2, Smad2*, *MafA*, and *Pdx1*(Figure 7A). Furthermore, immunostaining of Tg(*βmiR-21*) mouse islets concurrently showed decreased expression of multiple markers associated with β-cell identity and function, including MafA, Pdx1, Glut2, Pcsk1, and Pcsk2 (Figure 7B–E, Supplementary Figure 7, with quantification in 7I). An increase in Aldh1a3 expression was observed in the Tg(*βmiR-21*) mice (Figure 7F). Consistent with our results in INS1-miR-21 cells, Tg(*βmiR-21*) islets displayed decreased staining for Tgfb2 and Smad2 (Figure 7G–H).

### 3.6 Induction of miR-21 in human islets is associated with a dedifferentiated phenotype and reduced expression of miR-21 target mRNAs linked to β-cell identity

**Figure 8:**
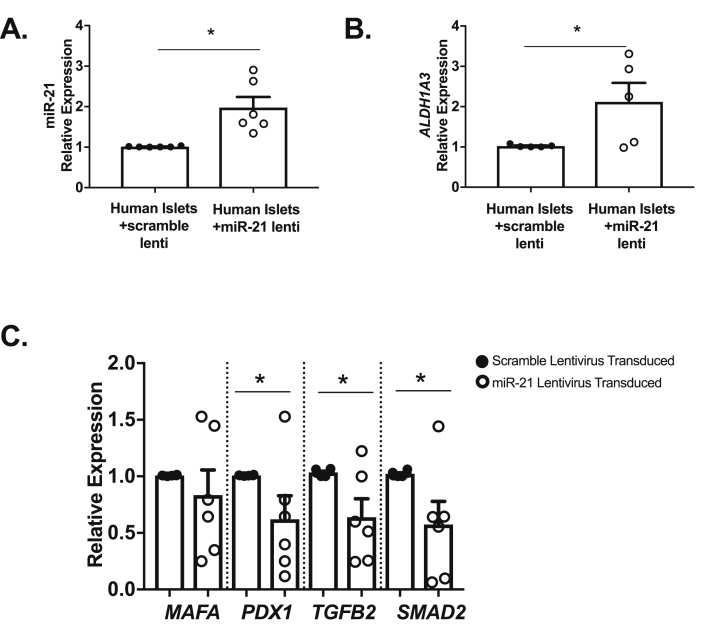
**Induction of miR-21 in human islets leads to a dedifferentiated phenotype and reduced expression of mRNAs regulating β-cell identity. (A)** miR-21 levels were increased in human islets transduced with the miR-21 virus as compared to human islets transduced with scramble virus **(B)** Levels of *ALDH1A3* are increased in miR-21 induced human islets. **(C)** mRNA expression of *PDX1,* along with *TGFB2* and *SMAD2* was significantly decreased in human islets transduced with the miR-21 lentivirus. n = 6; ∗p < 0.05.

To determine if observed miR-21 effects were relevant to human disease, human islets were transduced with miR-21 lentivirus to increase islet miR-21, or a scrambled control miRNA (Figure 8A; human donor details in Supplementary Figure 8 and Supplementary File 3). Islets from one donor (donor 4) did not exhibit changes in *Aldh1a3*, *Pdx1* or, *Mafa* mRNAs, despite successful overexpression of miR-21. However, even with the inclusion of this donor's data, consistent with our model systems *in vitro* and *in vivo*, miR-21 induction resulted in significantly increased *Aldh1a3* mRNA expression (Figure 8B) and significant reductions in *Pdx1,* along with *Tgfb2* and *Smad2* mRNA expression (Figure 8C).

## 4 Discussion

Prior studies have linked loss of β-cell identity and dedifferentiation to β-cell dysfunction in models of insulin resistance and reduced islet mass [38, 40]. Recent data have also identified this reversion to a progenitor-like state, as a compensatory response to islet inflammatory stress, with evidence of β-cell dedifferentiation in models of T1D [[38], [40]]. These studies have collectively characterized this phenomenon as follows: 1) downregulation of key transcription factors crucial for β-cell development, maintenance of identity, and function in association with upregulation of Aldh1a3 and progenitor markers; 2) inability to maintain a glucose-responsive state; and 3) exhibition of features of other islet endocrine cells [[35], [36], [37], [41], [42], [43], [44]]. Additionally, reduced prohormone processing enzyme expression has also been described in models of islet dedifferentiation, a phenotype observed in our models, with an increase in proinsulin relative to insulin staining, and decreased expression of processing enzymes [44]. A recent study identified gene signature profile changes in murine embryonic β-cells and adult β-cells following STZ treatment to identify β-cell dedifferentiation and dysfunction markers using single-cell RNA-seq [45]. Interestingly, an overlap analysis between the RNA-seq dataset generated by our miR-21 inducible INS1 cell line and the Sachs et al. dataset displayed an overlap of several statistically significant (FDR<0.5) genes (58 upregulated and 165 downregulated) (included in Supplementary File 4). Differences in this phenotype between studies likely reflect differences in terminology, differences in models used, and importantly, the likely existence of this phenomenon on a spectrum—with heterogeneous effects between disease states, individuals, or even between beta cells within islets.

As features defining loss of β-cell identity continue to be elucidated, the determination of underlying molecular mechanisms contributing to these effects is needed. Here, we identify a novel relationship linking cytokine-induced increases in β-cell miR-21 to reduced expression of mRNAs specifying β-cell identity and β-cell function. The sequence of miR-21 is highly conserved across multiple species including rats, mice, zebrafish, and humans (Supplementary Figure 9). This allowed for the usage of multiple model systems *in vitro* and *in vivo* to validate a conserved role of increased β-cell miR-21 in loss of β-cell identity, suggesting that this pathway could be an important physiologic response to islet inflammation.

Our results showed that miR-21 exerts effects on β-cell identity in part through direct targeting of mRNAs in the *Tgfb2* pathway. This signaling pathway has also been implicated in the development of the endocrine pancreas [13], β-cell development, and postnatal β-cell identity and function [14]. *Tgfb2* overexpression in rat islets *in vitro* increases insulin secretion [46]. Our data also suggest that increasing *Tgfb2* and *Smad2* partially abrogated miR-21's effects on mRNAs critical for β-cell function and identity. Prior work has shown that inhibition of *Tgfb2* and *Smad2* was associated with islet dedifferentiation [47]. By contrast, combined pharmacological inhibition of human β-cell DYRK1A and the TGF-β superfamily did not lead to a dedifferentiated phenotype [48]. These differences could potentially result from combined treatment, off-target effects of pharmacologic inhibitors, or the impact of miR-21 on multiple mRNAs.

Both zebrafish and mouse models of islet miR-21 induction displayed reduced expression of transcription factors specifying β-cell identity and in insulin^+^ cells, with increases in double positive insulin^+^ and glucagon^+^ islet cells, and hyperglycemia or glucose intolerance. However, there was a more drastic increase in double positive insulin^+^ and glucagon^+^ islet cells in the zebrafish compared to our mouse model. Differences in islet findings could be related to several differences in the model systems. The degree of β-cell miR-21 induction in zebrafish was higher than that observed in mice. Additionally, although we designed both systems to achieve post conception inducible miR-21 expression, because of the nature of our zebrafish model, miR-21 induction occurred 3-days post fertilization vs. 8-weeks after birth in the mouse model, which could impact effects on β-cell fate. Notwithstanding these differences, the overall similarities between our findings across model systems support the idea of miR-21 as a conserved modulator of β-cell identity.

The use of miRNA mimics can saturate RISC complexes and displace other endogenous miRNAs, causing disproportionately increased binding with lower affinity targets that may not be as dramatically impacted by lower level increases in the miRNA of interest [34]. Furthermore, mimic transfection yields overexpression of the predicted sense strand (5p strand) of the miRNA, while *in vivo* induction of pre-miRNA transcripts could lead to differential effects owing to activities of the antisense strand of the miRNA duplex [34]. In this study, to address this issue, we designed a lentiviral system of pre-miR-21 induction to model increases in pre-miR-21 on the scale of those observed in models of islet inflammation and diabetes [18].

A limitation of our study is the variability in observed impacts of miR-21 and TGFB/Smad signaling within and across our different systems, especially in human islet studies, where islets from one donor did not show an effect of miR-21 on β-cell identity, consistent with heterogeneity in human disease. Although inter-species differences in effects may exist, we consider the inclusion of multiple model systems a strength, and the fact that significant changes in features associated with β-cell identity are present across each of these systems is suggestive of a conserved physiologic response of the β-cell to increases in miR-21. Our GSIS in INS1 cells was not normalized for differences in cell death, which we previously observed on a larger scale in experiments performed using miR-21 mimics. However, lentiviral induction of more physiologic increases in miR-21 appeared to have a differential effect on β-cell function; consistent with this, mimic experiments associated with larger increases in beta cell apoptosis demonstrated higher insulin secretion at baseline for miR-21-5p mimic-transfected cells, with no increase in insulin secretion following high glucose treatment [6]. In contrast, in the present study, baseline and stimulated GSIS were both reduced in concert.

In conclusion, these studies have defined a new mechanism that links increases in β-cell miR-21 to β-cell dysfunction during diabetes development. Furthermore, our study has identified a novel upstream molecular modulator of β-cell identity, and a mechanistic pathway initiating β-cell dedifferentiation in the context of islet inflammatory stress. The use of several model systems and human islets ensure that these results are robust and relevant to human diabetes. Future studies should test the potential for therapeutic targeting of islet miR-21 and its molecular signaling pathways to preserve functional β-cell mass in diabetes.
