# Supplementary material for: β-Cell pre-mir-21 induces dysfunction and loss of cellular identity by targeting transforming growth factor beta 2 (Tgfb2) and Smad family member 2 (Smad2) mRNAs
Source: Mol Metab. 2021 Jul 9;53:101289. doi: 10.1016/j.molmet.2021.101289 (PMC8361274; doi:10.1016/j.molmet.2021.101289)
Supplement: Supplementary file 5 — Multimedia component 5 [file mmc5.docx]

Online Supplemental Materials

**B.**


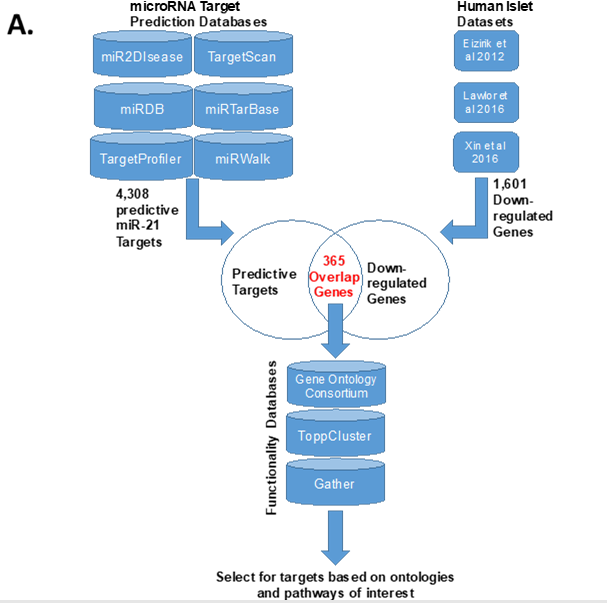

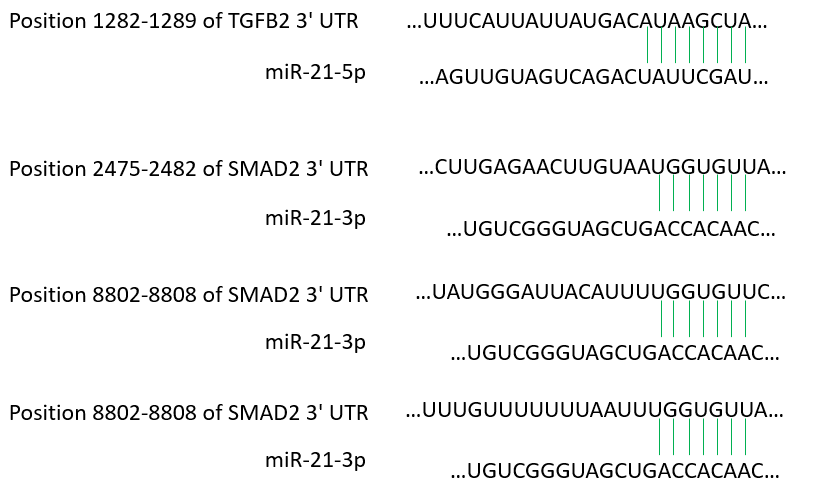


**Supplementary Figure 1: A comprehensive informatics analysis indicates that β cell miR-21 is involved in cell commitment processes.** **(A)** Workflow for determining β cell miR-21 targets. A search of 6 miRNA target prediction databases yielded 4,308 unique predicted miR-21-5p or -3p targets (14-18). Predicted mRNA targets were cross-referenced with publicly available RNA Sequencing datasets from β cells of T2D versus non diabetic human islets (19; 20) and human islets treated with a cytokine cocktail compared to untreated control islets (21). This yielded 365 potential miR-21 target mRNAs that are impacted during diabetes and inflammatory stress. Ontology and pathway analysis was then performed using functionality databases (22; 23). **(B)** miR-21 is predicted to bind to *Tgfb2* and *Smad2* mRNAs. Predictive binding sites of miR-21 within the 3’ Untranslated regions (3’UTRs) *ofTgfb2* and *Smad2* are delineated.

**Supplementary Figure 2: miR-21 levels can be induced in INS1-miR-21 cells in a dose dependent manner.** Levels of pre-miR-21 were measured via RT-PCR in response to increasing doses of Doxycycline in INS1-miR-21 and INS1-Scramble cells.

**
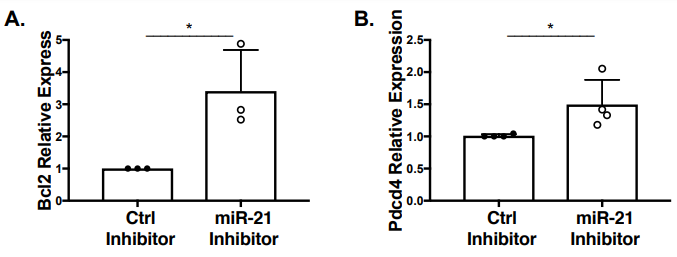
**

**Supplementary Figure 3: Inhibition with a miR-21 inhibitor leads to increased levels of Bcl2 and Pdcd4 transcripts.** Expression of Bcl2 and Pdcd4 mRNAs was measured via RT-PCR. n=3-4; *p<0.05.


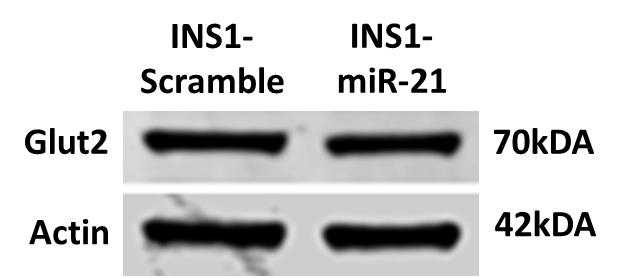


**A.**


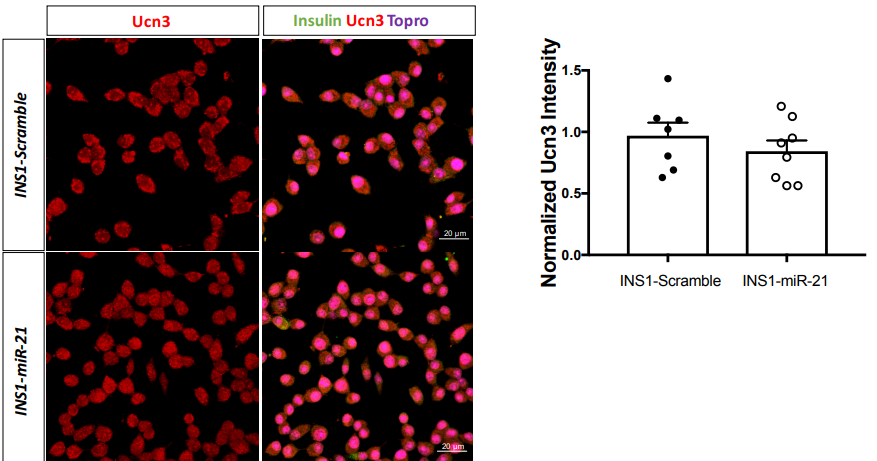


**B.**

**Supplementary Figure 4: INS1-miR-21 cells did not have a decrease in Glut2 and Ucn3 protein levels. (A)** No changes in protein levels of Glut2 were detected by western blot analysis (n=1). **(B)** No measurable changes were appreciated between INS1-Scramble and INS1-miR-21 cells (n=7-8).


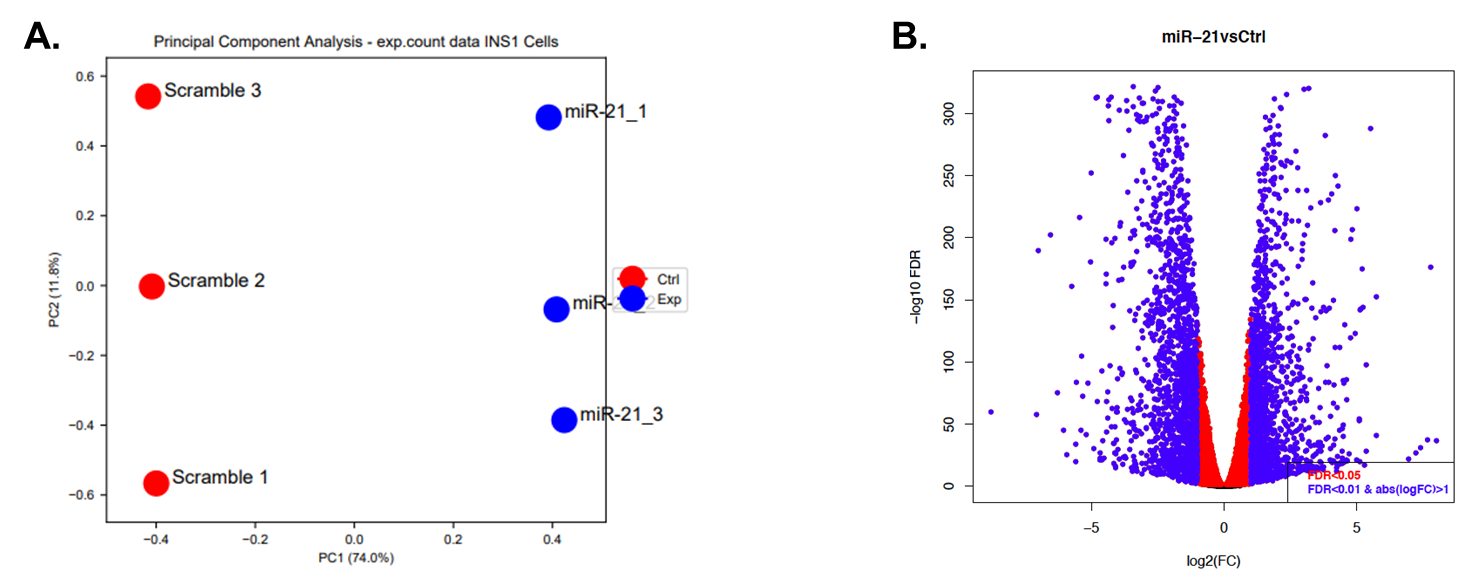


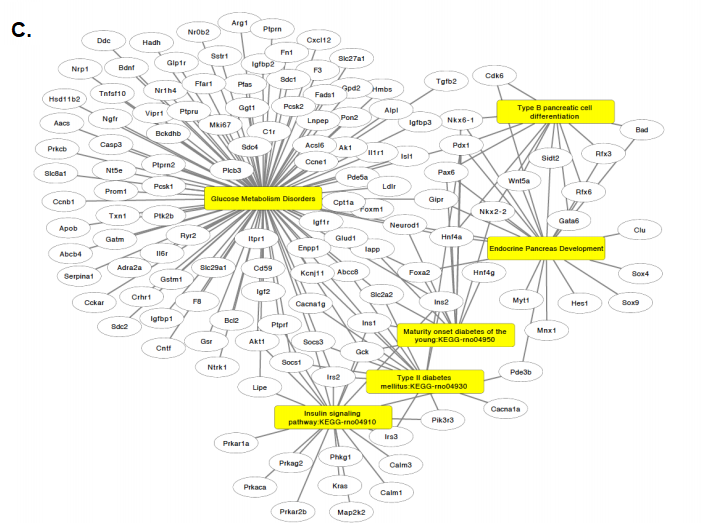


**Supplementary Figure 5:** **RNA sequencing of INS1-miR-21 cells.** (**A)** MDS analysis for samples. **(B)** Volcano plot shows genetic differences between the two groups. **(C)** Network analysis of ontologies and pathways associated with downregulated genes in the dataset generated from sequencing INS1-miR-21 cells and INS1-Scramble control cells. n=3.

**A.**

**B.**

**Supplementary Figure 6: There is no difference in IPGTT between the two control groups and levels of miR-21 are not increased in other tissues in the Tg(βmiR-21) mice. (A)**Two different control groups were used to compare against the Tg(βmiR-21) mice: Cre^+^LACZ^-^ mice and Cre^-^LACZ^+^ mice. These two groups show no difference in blood glucoses, indicating that phenotypic differences observed are not Cre dependent. The genetic background for all mice used in this experiment is C57BL/6. An F1-F3 generation of littermates was used for experimental data collection. **(B)** Levels of miR-21 were measured in brain, small intestine, skeletal muscle, fat, and liver of Tg(βmiR-21) mice and littermate controls. Levels of miR-21 were not increased in any tissues as compared to littermate controls.

**A.**


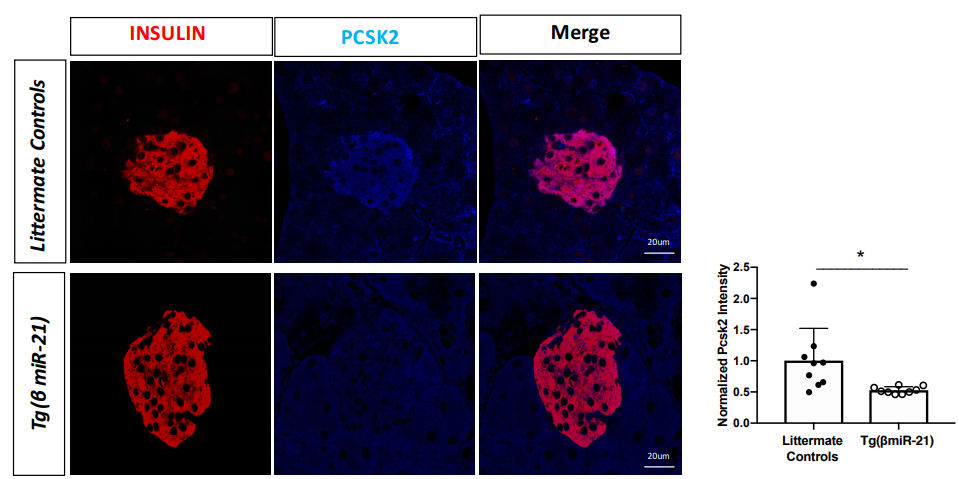


**Supplementary Figure 7:** Tg(*βmiR-21*) mice display decreases in β cell markers. Decreased expression of Pcsk2 was observed in insulin+ cells in Tg(*βmiR-21*) mice. n=9; *p<0.05.


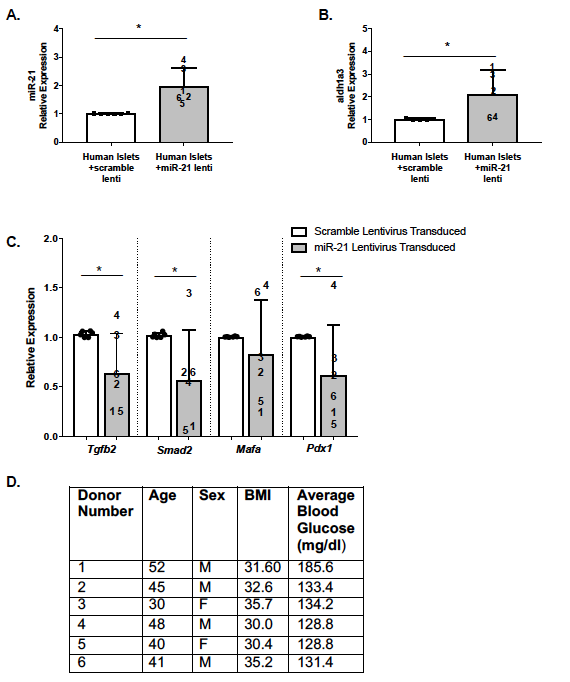


**Supplementary Figure 8: Donor information corresponding to induction of miR-21 (A)** miR-21 levels were increased in human islets transduced with miR-21 virus as compared to human islets transduced with scramble virus n=6; *p<0.05. **(B)** Levels of *Aldh1a3* are increased in miR-21 induced human islets n=6; *p<0.05. **(C)** Targets of interest are decreased within human islets transduced with miR-21 lentivirus. Levels of *Tgfb2, Smad2,* and *Pdx1* are decreased in human islets transduced with miR-21 lentivirus n=6; *p<0.05. **(D)** Donor information corresponding to each point on the graphs.

**Supplementary Figure 9: miR-21 is highly preserved across multiple species.** The mature miR-21 sequence, including the predictive active seed sequence, is highly preserved across humans, mice, rats, and zebrafish.

**Supplementary Table 1: Primers used for analysis.**

| **Rat Primers Used:** | |
| --- | --- |
| **Primer name** | **Primer sequence** |
| NKX6.1_FWD | ACTTGGCAGGACCAGAGAGA |
| NKX6.1_REV | GGGCTTGTTGTAATCGTCGT |
| Aldh1a3_FWD | GGGTCACACTGGAGCTAGGA |
| Aldh1a3_REV | CTGGCCTCTTCTTGGCGAA |
| Tgfb2_FWD | GATCTAACCTGTTGCCTGTGT |
| Tgfb2_REV | CCATGTATCTCGCTGTTCCC |
| Smad2_FWD | ACCATAAGAATGAGCTTCGTG |
| Smad2_REV | GTTAATACTTTGTCCAACCACTGC |
| Mafa_FWD | CTTCAGCAAGGAGGAGGTCATC |
| Mafa_REV | GCGAGCCGCGGTTCTT |
| Pdx1_FWD | GATGAAATCCACCAAAGCTCAC |
| PdX1_REV | GTACGGGTCCTCTTATTCTCCT |
| NeuroD1_FWD | AGCTCCCACGTCTTCCACGTC |
| NeuroD1_REV | GGCTTTCAAAGAAGGGCTCCAG |
| Nanog_FWD | AGCCTCCAGCAGATGCAAGAA |
| Nanog_REV | TTGCACTTCATCCTTTGGTTTTGA |
| Glut2_FWD | CTGGGTCTGCAATTTCATCA |
| Glut2_REV | CGTAAGGCCCGAGGAAGT |
| Ins2_FWD | CTACAATCATAGACCATCAGCA |
| Ins2_REV | CAGTTGGTAGAGGGAGCAGAT |
| Pcsk2_FWD | ACACAGCTCCGCACATTCGCA |
| Pcsk2_REV | TGAGATCCACAACCGCCCTCCA |
| Pcsk1/3_FWD | AGTTGGAGGCATAAGAATGCTG |
| Pcsk1/3_REV | GCCTTCTGGGCTAGTCTGC |
| PCNA_FWD | TAAAGAAGAGGAGGCGGTAA |
| PCNA_REV | TAAGTGTCCCATGTCAGCAA |
| Gck_FWD | CAACTGGACCAAGGGCTTCAA |
| Gck_REV | TGTGGCCACCGTGTCATTC |
| Ngn3_FWD | CTGCGCATAGCGGACCACAGCTTC |
| Ngn3_REV | CTTCACAAGAAGTCTGAGAACACCAG |
| Ins1_FWD | TAGTGACCAGCTATAATCAGAG |
| Ins1_REV | ACGCCAAGGTCTGAAGGTCC |
| L-Myc_FWD | CCATCAGCAACAGCACAACTA |
| L-Myc REV | CACTTTCTACAGGTGGGGGA |
| **Zebrafish Primers Used:** | |
| Tgfb2_FWD | GCGCTTTGCAGGTATAGACG |
| Tgfb2_REV | TGGCTCTTATGCTGCGACTC |
| Pdx1_FWD | CACGGTTTCCCCGGTCTATG |
| Pdx1_REV | GACACGGTGGAGGGTTTTGG |
| Smad2_FWD | TTCACAGACCCCTCCAATTC |
| Smad2_REV | GATGGCGCTATCGCTAAGAC |
| Mafa_FWD | ATCACCAGCACAGCCACCTG |
| Mafa_REV | TTGAGCCGGATCACCTCCTC |
| miR-21_FWD | GGTTATGTGTCTTTATTGGCGT |
| Universal REV | GTGCAGGGTCCGAGGT |
| **Mouse Primers Used:** | |
| Aldh1a3_FWD | GGGTCACACTGGAGCTAGGA |
| Aldh1a3_REV | CTGGCCTCTTCTTGGCGAA |
| **Human Primers Used:** | |
| Tgfb2_FWD | TGCTTTGGCTTTCTGGTTCT |
| Tgfb2_REV | TTTGTTTGTGGTGCAGTGGT |
| Smad2_FWD | ACTAACTTCCCAGCAGGAAT |
| Smad2_REV | GTTGGTCACTTGTTTCTCCA |
| Mafa_FWD | ATTCTGGAGAGCGAGAAGTGCCAA |
| Mafa_REV | CGCCAGCTTCTCGTATTTCTCCTT |
| Pdx1_FWD | TACTGGATTGGCGTTGTTTGTGGC |
| Pdx1_REV | AGGGAGCCTTCCAATGTGTATGGT |
| Aldh1a3_FWD | TCACAGACAACATGCGGATT |
| Aldh1a3_REV | TCGGTGCTATTCGCTCTTTT |

Primers used for detecting miR-21 levels in mouse, human and rat samples were from Qiagen (miScript Primer assays for pre-miR-21).

**Supplementary Table 2: Antibodies used for analysis**

| **Antibody name and company:** | **Host species:** | **Antibody dilution** |
| --- | --- | --- |
| **Antibodies for Western Blot Analysis:** |  |  |
| Smad2: Cell Signaling | Rabbit | 1:500 |
| Pdx1: Cell Signaling | Rabbit | 1:1000 |
| Mafa: Sigma | Rabbit | 1:500 |
| Tgfb2: R&D | Mouse | 1:250 |
| **Antibodies for staining:** |  |  |
| Aldh1a3: Novus | Rabbit | 1:200 for zebrafish and 1:500 for mouse samples |
| Insulin: Invitrogen | Guinea Pig | 1:200 for zebrafish and 1:500 for mouse samples |
| Glucagon: Sigma | Mouse | 1:200 for zebrafish and 1:500 for mouse samples |
| Tgfb2: R&D | Mouse | 1:50 for zebrafish and mouse |
| Smad2: CST | Rabbit | 1:50 for mouse |
| Nkx6.1: DSHB | Mouse | 1:50 for zebrafish |
| Neurod1: abcam | Rabbit | 1:50 for mouse |
| Pcsk2: Novus | Mouse | 1:50 for mouse |
| Gck: R&D | Mouse | 1:50 for mouse |
| Glut2: Novus | Rabbit | 1:50 for mouse |
| Ngn3: LS Bio | Rabbit | 1:50 for mouse |
| Nanog: BioLegend | Mouse | 1:50 for mouse |
| Ki-67: Novus | Rabbit | 1:50 for mouse |
| L-Myc: Abcam | Rabbit | 1:50 for mouse |
| PH3: Sigma | Rabbit | 1:100 for mouse |
| Pcsk1: CST | Rabbit | 1:50 for mouse |
